# Introduction to the Course of Lectures on Diseases of the Eye, in Rush Medical College, Session of 1866–7

**Published:** 1867-01

**Authors:** E. L. Holmes

**Affiliations:** Surgeon to the Chicago Charitable Eye and Ear Infirmary, Etc.


*Introduction to the Course of Lectures on Deseases of the Eye, in Rush Medical College, Session of 1866–67.* By E. L. HOLMES, M.D., Surgeon to the Chicago Charitable Eye and Ear Infirmary, etc.

GENTLEMEN—Before we enter upon the actual investigation of the subjects which we propose to discuss in this course of lectures, I am desirous of calling your attention to a few practical points in reference to your studies here as students, and to your future labors as practitioners.

I do not wish to magnify the importance of ophthalmology; the importance of the organ itself, of which it treats, the great suffering so often caused by its manifold diseases, and the terrible calamity which befalls the unfortunate patient when its diseases have forever extinguished the sense of sight, render it unnecessary to urge its claims especially upon you.

If it is the great object of the physician to prolong life, to preserve the health of every organ in the human system, and to prevent physical and mental suffering, there would seem to be scarcely a reason why the affections of an organ so delicate and useful, and so liable to disease, as the eye, should ever be so generally neglected by those who are preparing themselves for the duties of our profession.

It is stated on good authority, that one-twelfth of all the patients treated in the English hospitals suffer from affections of the eye. In this country, there is probably about the same proportion of ophthalmic diseases. Physicians in certain portions of this State, in active general practice, have informed me that at times one-third of their patients are afflicted with diseases of the eye.

And yet, it is true that the study of these diseases and their treatment have been almost absolutely neglected, not only by the student of medicine, but by the majority of practitioners. The causes which have tended to detract the attention of the profession from ophthalmology are to be found in the great obscurity which has existed, regarding the nature and treatment of diseases of the eye, and in the absence of instruction in our medical colleges, in consequence of the short term of study required previous to the examination for the degree of medicine. Practitioners too often fail to pursue in this department of their education what they omitted in their collegiate course.

Discredit has been thrown upon this special department, from the fact that the treatment of diseases of the eye has been left to such an extent in the hands of incompetent and unprincipled charlatans. When such a field of labor was abandoned, as it were, by educated practitioners, it is scarcely a matter of surprise that it should have been occupied by ignorant pretenders. So much light, however, has been thrown upon our knowledge of ophthalmic diseases; so many valuable works on the subject have been recently presented to the profession; the facilities for teaching this branch have been extended to such an extent in our schools; the infirmaries for the special treatment of diseases of the eye in several of our large cities afford so large a number of cases for clinical instruction, that extraordinary interest in ophthalmic science has been developed in this as well as every other country.

I apprehend very much that is said regarding the importance of ophthalmology is called forth in consequence of the very great neglect which the science has so long suffered. Undoubtedly, if diseases of the lungs, or if the treatment of fractures, or any class of affections, had been almost absolutely omitted in the instruction of our medical colleges, with the expectation that the student could secure the necessary knowledge after he had entered upon active practice, there would be the same discussion regarding the importance of the study of these affections, as now in regard to the study of diseases of the eye.

You can, with your best efforts, but lay the foundation of a medical education during your college courses. It is for your interest that this foundation should be broad and thoroughly laid. Your medical studies, therefore, in this place, should have scarcely any direct reference to a specialty; for no specialty can be cultivated with full advantage without a good knowledge of every important branch of medical science. To secure this knowledge, requires at least three years of most careful, unremitted study. Even this peroid is scarcely sufficient to prepare the student for the responsibilities of actual practice.

It should be remembered that, almost without exception, those who have distinguished themselves as specialists in any department have been thoroughly educated physicians. I therefore most earnestly recommend you all to follow sedulously the lectures of each of your teachers, and read upon each subject all your time will permit. Those of you, whose sphere of labor will be in a thinly settled country, should not, under ordinary circumstances, devote your time to any specialty. The people around you depend upon you for relief in every form of disease and accident. You must be their physician, surgeon, obstetrician, oculist, aurist, and, to a certain extent, their dentist; you should, therefore, comprehend the affections of every organ. Neither your time and opportunity for observation, nor the demands of the community in which you may practise, will permit you to cultivate a specialty with advantage.

But as you advance in years and in experience, and you become surrounded by a denser population, you can then, perhaps with advantage to yourselves and your patients, follow your tastes in the study of special diseases.

Those of you, on the other hand, who will enter upon practice in a thickly settled town or city, will be placed in a somewhat different position. You can then restrict your labors to such a branch or branches of our profession as your tastes or as accidental circumstances may dictate. There will be a sufficient number of practitioners near you to preclude all danger that the sick cannot secure immediate and prompt attention.

To those of you who have not finished your collegiate course, I would, therefore, give this advice:—Attend the lectures on diseases of the eye at the clinic and lecture room, and study, as you find opportunity, a suitable text-book, precisely as you do in the other branches taught in this place by your different professors. Make it your chief object in this, as in other departments, to obtain those fundamental principles which, if fully comprehended, will render your future studies comparatively easy, in whatever direction you wish to pursue them.

And yet, I would have you distinctly understand that I am a warm advocate of the subdivision of professional labor, provided this subdivision is based upon correct principles. I believe any system of education is erroneous, which tends to restrict the student in college to the investigation of one class of diseases. Each organ of the body is so intimately connected with the other organs, that the skilful diagnosis and treatment of its diseases require a range of study far more extensive than that of its anatomy, physiology, pathology, and therapeutics. No one can be a good oculist without being a good physician.

It is true, the field of labor in the science of medicine is so extensive that no one can ever hope to attain perfection in all its departments. It is fallacious, however, to maintain that, because an intimate knowledge of the whole science of medicine is beyond the capacity of any intellect, one can become really perfect in one department by neglecting all others. The pursuit of a specialty, to the neglect of any subject practically related to the science of medicine, cannot fail to narrow the mind and render it less capable of thoroughly investigating the disease even of one organ.

Be determined, therefore, if you intend to devote your best energies to the study of ophthalmology, first, to make a careful investigation of every branch of medical science; endeavor to become familiar, as general practitioners, with all diseases; then, as circumstances become favorable for such a step, relinquish the practice, for instance of surgery and obstetrics, and devote more and more of your time to diseases of the eye.

Our present course, as you well know, will consist of a comparatively few lectures in this place, and of two clinics each week at the Eye and Ear Infirmary. It will be my chief object to direct you in entering upon the study of ophthalmology, and to excite such an interest in the subject that you will give it the attention it deserves.

As text-books for you, as beginners, I would recommend for your careful perusal, the recent work of Mr. Laurence and Mr.

Moon, and the two small but excellent volumes of Dr. Williams, of Boston. After the principles of the science, presented in either of these works, have been carefully impressed on your minds, and you have examined the cases at the infirmary requiring surgical or medical treatment, you will be, to say the least, prepared to commence and fully comprehend any of the standard works you choose to study.

Prof. Stellwag, of Vienna, has undoubtedly written the most valuable text-book on ophthalmology, which can be found in any language. It will, probably, be soon translated into English, and will be a work you should all possess.

The work of Wecker, of Paris, not yet completed, will be a most valuable contribution to ophthalmic literature. At the present time, there is not a single manual on diseases of the eye in the English language corresponding, for instance, to the work of Prof. Flint, on general medicine.

There are, however, many valuable monographs and two very important ophthalmic journals:—The *Ophthalmic Review*, published semi-annually in London, and edited by Laurence and Winsor; and the *Royal London Ophthalmic Hospital Reports*, edited by Wordsworth, Hulke, and Hutchinson. These journals are invaluable, and worthy of your attention.

Those of you who read German, will find two journals whose pages are filled with most interesting matter, not only on theoretical but practical subjects connected with ophthalmology. One is edited by Arlt, Donders, and Græfe; the other by W. Zehender.

For an ample discussion of the principles and application of the ophthalmoscope, I must refer you to the work of Zahnder, translated in England by Carter, and soon to be reprinted in this country. This, in connection with the beautiful ophthalmoscopic plates of Jæger and Liebreich, will enable you, with practice in examinations with the ophthalmoscope, to understand the abnormal appearances you may detect in the optic nerve (papilla), retina, choroid, and vitreous humor.

You will find in the celebrated work of Donders, translated by Dr. Moore, for the New Sydenham Society, nearly all that is known regarding the accommodation of the eye for vision at different distances, the abnormal conditions of the refracting media, the causes of strabismus, and the use of glasses. This work, although very large, is so divided in its chapters, that the "practical" portions are entirely distinct and separate from what might be termed by many the theoretical and comparatively useless portions.

I cannot close this lecture, without urging upon you the following important advice:—Form the habit of frequently examining the eye in health in persons of different ages and different constitutions; learn to observe at a glance the condition of the palpebral conjunctiva, and of the margin of the lids, and the position of the puncta and ciliæ; the form, size, (apparent) color, and movability of pupil; the color and position of the iris and the surface of the cornea, as seen with the light falling upon it from various directions.

By cultivating this habit, you will be able to recognize the difference between the conditions of health and disease much more readily than you can with no positive knowledge of the various normal appearances of the eye.

